# Networks of Twin Peaks: The Dale Cooper Effect

**DOI:** 10.1007/s00283-022-10187-w

**Published:** 2022-06-01

**Authors:** Harun Siljak

**Affiliations:** Trinity College, Dublin, Ireland

What mathematical object, if any, do you imagine when you read/watch/listen to a story? This article was inspired by Kurt Vonnegut’s and Ursula K. Le Guin’s views on storytelling and by the creative work of David Lynch and Mark Frost on the TV show *Twin Peaks*. It is worth saying right at the start that no prior knowledge of any of this is needed for reading this article.

## The Shape of Stories

**Figure 1.**
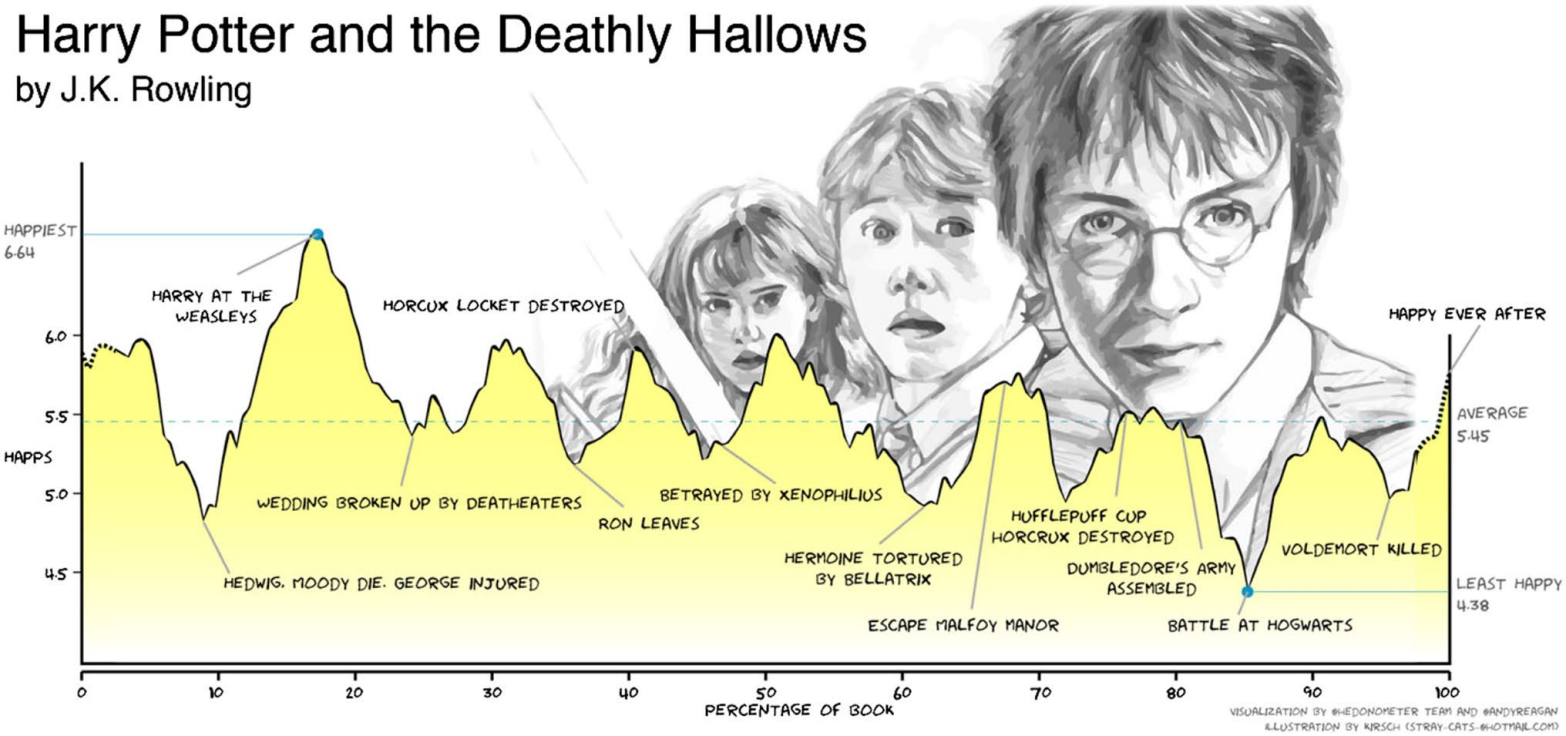
Annotated emotional arc of Harry Potter in *Harry Potter and the Deathly Hallows*, by J. K. Rowling. (Reproduced from [8] under Creative Commons Attribution 4.0 International License.)

When Kurt Vonnegut gives his humorous account of the shape of stories [11], he is in the realm of calculus, continuous functions describing dynamics of the plot, the highs and lows of the protagonist’s emotions, the speed at which the story unravels. In a way, it corresponds to the historical development of calculus as a way to interpret motion as a function of time. The abscissa is the time, and the ordinate axis is something important for the hero, his emotional state, for example, as shown in Figure 1, which provides a detailed illustration from contemporary fantasy fiction taken from [8], in which the authors performed a systematic quantitative analysis of Harry Potter’s emotional ups and downs.

There is another shape of a story in which we are interested—that of its characters, the world in which they live. That shape focuses not on the story’s actions, but on its structures. It describes the contents of the carrier bag, as in Ursula K. Le Guin’s “The Carrier Bag Theory of Fiction” [4], and those contents can be neatly arranged in graphs, maps, and trees (as the thus-named work by Franco Moretti suggests [6]). Its tools come from graph theory and algebra, and once put in the context of arbitrarily large networks, the field of research is usually called network theory [1].

Are there only so many community types and networks of interactions in life and in literature? What can mathematics tell us about this? Are networks of characters in fiction anything like our social networks? A growing body of literature has been dealing with this application of network theory, investigating epic stories from the past, superhero universes from the modern era, etc. The greater the number of characters in a work of fiction, the more interesting and relevant network-theoretic results become: patterns emerge as clusters and communities, and unexpected characters reveal themselves as keystone elements of networks. If they are removed, everything falls apart.

In the following quotation from Le Guin lies our motivation to explore such networks, rather than the forward-driving, sharp-edged plots of Vonnegut:

I would go so far as to say that the natural, proper, fitting shape of the novel might be that of a sack, a bag. A book holds words. Words hold things. They bear meanings. A novel is a medicine bundle, holding things in a particular, powerful relation to one another and to us [4, p. 6].

She continues:

Finally, it’s clear that the Hero does not look well in this bag. ... That is why I like novels: instead of heroes they have people in them.

We are curious as to what the hero effect is in a network: are heroes just like anyone else, do they fit in the bag? Finally, we erase the linear time of Vonnegut’s plots by observing networks instead of actions, algebra instead of calculus. The community in the network exists independently of the passage of time, and it needs to feel real. Again, Le Guin writes:

If, however, one avoids the linear, progressive, Time’s-(killing)-arrow ... one pleasant side effect is that science fiction can be seen as ... in fact less a mythological genre than a realistic one [4, p. 7].

In this work, we scratch the surface of character networks in the cult TV show *Twin Peaks*. For readers who have not seen it, the first season of *Twin Peaks* revolves around a murder investigation in the town of Twin Peaks, Washington. FBI agent Dale Cooper comes to town to investigate alongside the local police department, and viewers get to see the entanglement of networks of crime, romantic relationships, and law enforcement along with a blurring of the lines between them. Compared to those in other network-theoretic treatises, this network is of small scale, with sixty characters introduced over the course of eight episodes. The community is not even expected to behave as a social network; the crime-investigation-driven plot of *Twin Peaks* was expected merely to shine a light on those relationships relevant to the murder of Laura Palmer, hyperfocusing on a network built by the law enforcement officers conducting the investigation. Not only did the network analysis confirm that the community is more than just a set of photos pinned to a plywood board at the Sheriff’s office; we also discovered a new storytelling network phenomenon I have called the Dale Cooper effect, a phase transition in network structure.

## Graphs and Associated Tools

**Figure 2.**
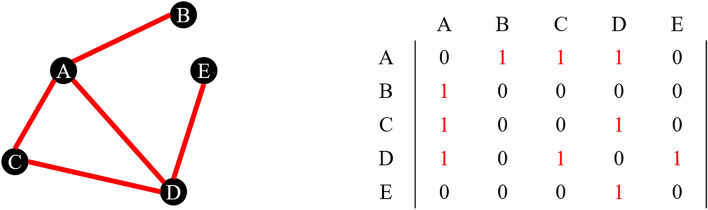
A graph and its adjacency matrix.

A graph is a mathematical object consisting of vertices and edges (Figure 2, left), which in the context of networks are often called nodes and links, respectively. A pair of vertices can be connected by a single edge or no edges, e.g., B and E have no edge connecting them, while A and B do. For large graphs and their algebraic manipulation, a commonly used representation is that of the adjacency matrix (Figure 2, right). It is a square matrix whose dimension corresponds to the number of vertices in the graph. As the figure shows, if there is an edge between the vertex corresponding to a row and the vertex corresponding to the column, we place a 1 at their intersection in the matrix. Otherwise, that element is a 0 (no edge). If a graph has no loops (i.e., if connecting vertex A to itself via an edge is not an option), the diagonal elements of the adjacency matrix are all zero. Furthermore, if the graph is undirected (i.e., if an edge between A and B is interpreted both as a connection of A to B and B to A), the matrix is symmetric.

**Figure 3.**
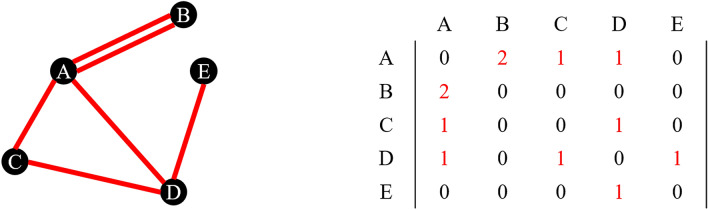
A multigraph and its adjacency matrix.

An extension of the concept of the graph is that of the multigraph, in which multiple edges connecting the same two vertices are allowed. An example of this is shown in Figure 3, where vertices A and B now have two common edges. This situation maps directly into the adjacency matrix, and the AB and BA entries are now equal to 2. This multigraph can also be interpreted in terms of weighted graphs: all single edges are considered to have weight 1, but a double edge, like that between A and B, will have weight 2. Sometimes, for example in collaboration networks, used to represent the distance between entities represented by nodes (e.g., coauthors in mathematics or costars in movies; cf. Erdős number, Bacon number), *n* multiple links between two nodes can be taken to correspond to the weight 1/*n*.

At this point, it is useful to define the degree of a vertex in a graph: for undirected graphs, it is simply the number of edges connected to it (i.e., the sum of the entries in the corresponding row or column in the adjacency matrix). In the example in Figure 2, the degree of nodes A and D is 3, the degree of node C is 2, and the degree of nodes B and E is 1.

In drawing conclusions from networks appearing in the world around us, it is often useful to compare their structure to that of basic network models; common features stand out and cross over in different contexts, suggesting general patterns. Two common random network models are the Erdős–Renyi (random network) and the Barabási–Albert (preferential attachment) models.

In the Erdős–Renyi model, the adjacency matrix is filled by tossing a biased coin. Let it be a $200\times 200$ matrix, and let the probability of two vertices being connected by an edge be 0.1. The upper triangle of the adjacency matrix is then populated entry by entry with the result of tossing a coin that has a 10% chance of heads (1) and a 90% chance of tails (0). After the upper triangle is filled, it is symmetrically copied into the lower triangle, finalizing the undirected Erdős–Renyi graph generation.

The Barabási–Albert model recognizes the observation that many networks in the real world are built with the mechanism of preferential attachment. When a new node is added to such a network, the links it forms are not drawn with equal probability from the set of all nodes already in the network. The nodes that are already well connected with other nodes will have a greater probability of attracting the new node. Let our network once again have 200 nodes; in the process of preferential attachment we will add one node at a time, assign four links connecting it to existing nodes in the network, and end the process once all 200 nodes are added. The probability that the newly added node will establish a link with a particular existing node is proportional to the existing node’s degree.

**Figure 4.**
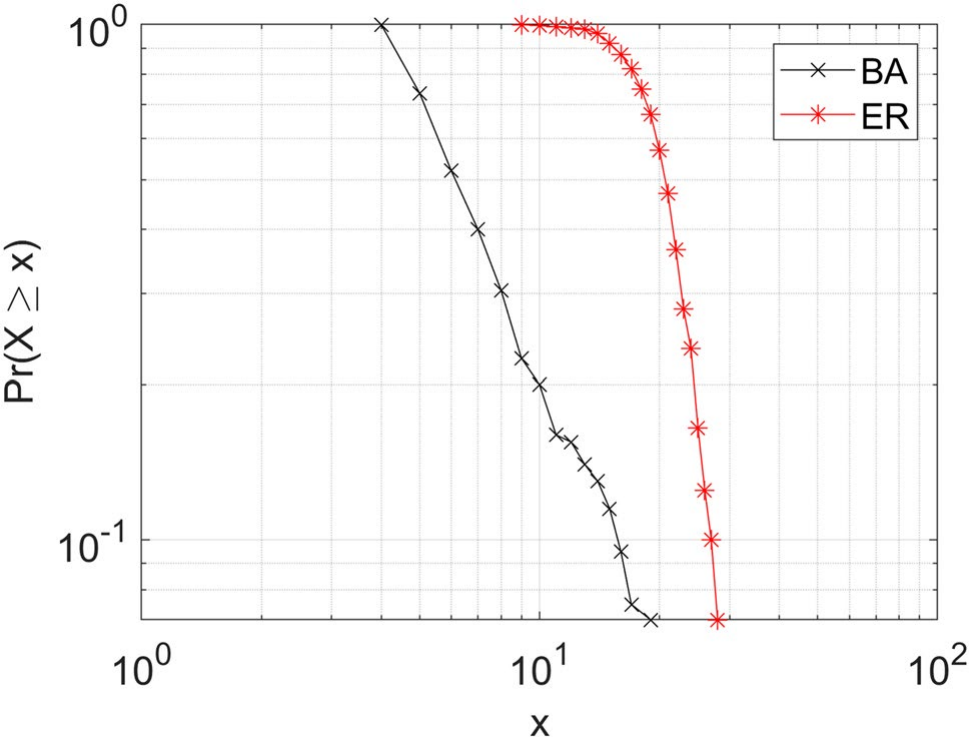
Examples of degree distributions for random networks.

In Figure 4 we depict the complementary cumulative distribution function of the degrees in the two network model examples we described. The plot represents the probability that a node in the network has degree *X* greater than some value *x*. For an *n*-node random network, the degree of a node is a function of $n-1$ coin tosses and hence is a function of scale: 50% of the nodes will be expected to have, in our numerical example, degree less than $200\cdot 0.1=20$, and 50% will be expected have a greater degree. However, for the preferential attachment network, no such scale exists, since the network generative process could have continued beyond *n* nodes (this is why we often refer to these networks as scale-free). The complementary cumulative distribution function for such a network is theoretically a straight line in a log-log plot (here we present the actual plot from a 200-node network to show the deviations seen at small scales for a single instance of a network). While the “preferential attachment results in power law” maxim often holds, interesting exceptions have been observed—a famous one is the citation distribution of *Physical Review* papers, demonstrated by Sid Redner to follow a log-normal distribution [9].

Another measure of interest for us in this note is that of assortativity. Are nodes with large/small degrees more likely to be connected to other vertices with large/small degrees in a network? In real-world social networks, it is expected that the more popular nodes of the network will cluster together, while in networks such as the internet, webpages with few links to other webpages are more likely to be connected to well-linked hubs. The former is the case of a positive assortativity coefficient, and the latter of a negative one. Random networks, such as those created by processes introduced earlier in this section, are expected to have a neutral (zero) assortativity.

A frequent reason for investigating networks in works of fiction is to compare their statistical properties to those of real-world networks. For example, in [5], the authors apply network theory tools to study networks of characters in the epic narratives *Beowulf*, *Táin Bó Cúailnge*, and the *Iliad*. The network of the *Iliad* was found to have features of realistic networks such as positive assortativity and scale-free behavior. For *Beowulf*, these properties are achieved once the protagonist, Beowulf, is removed from the story—Beowulf’s disproportionate degree of interaction with the world skews it. For *Táin Bó Cúailnge*, the key to reaching realistic network properties has been removing one-off interactions between the six protagonists of the epic and the remainder of the world. There is always a component of “historicity evaluation” in discussing the studies of epics; assessing the verisimilitude of their social networks is a factor in thinking about their genesis. For *Twin Peaks*, we have no such concerns; it is a fantasy world that we do not expect to be a snapshot of society. However, we are curious to see what the storytelling structure reveals.

## Twin Peaks

To investigate the networks of interaction in *Twin Peaks*, we analyzed the transcripts from season 1 of the show (total of eight episodes) [10]. The choice to restrict the dataset to season 1 was motivated by the evolution of the script and cast in season 2, followed by the constraints that the passage of time put on season 3 (filmed two decades after the first two). Season 1 was hypothesized to be the smallest homogeneous unit appropriate for analysis. The adjacency matrix was formed chronologically: every time a new character appears, a new node is added to the graph. For every scene in which two characters share screen time, a new edge is added to the graph, allowing multiple edges between the same pair of nodes. If three characters appear in the scene, all pairs get a new edge.

**Figure 5.**
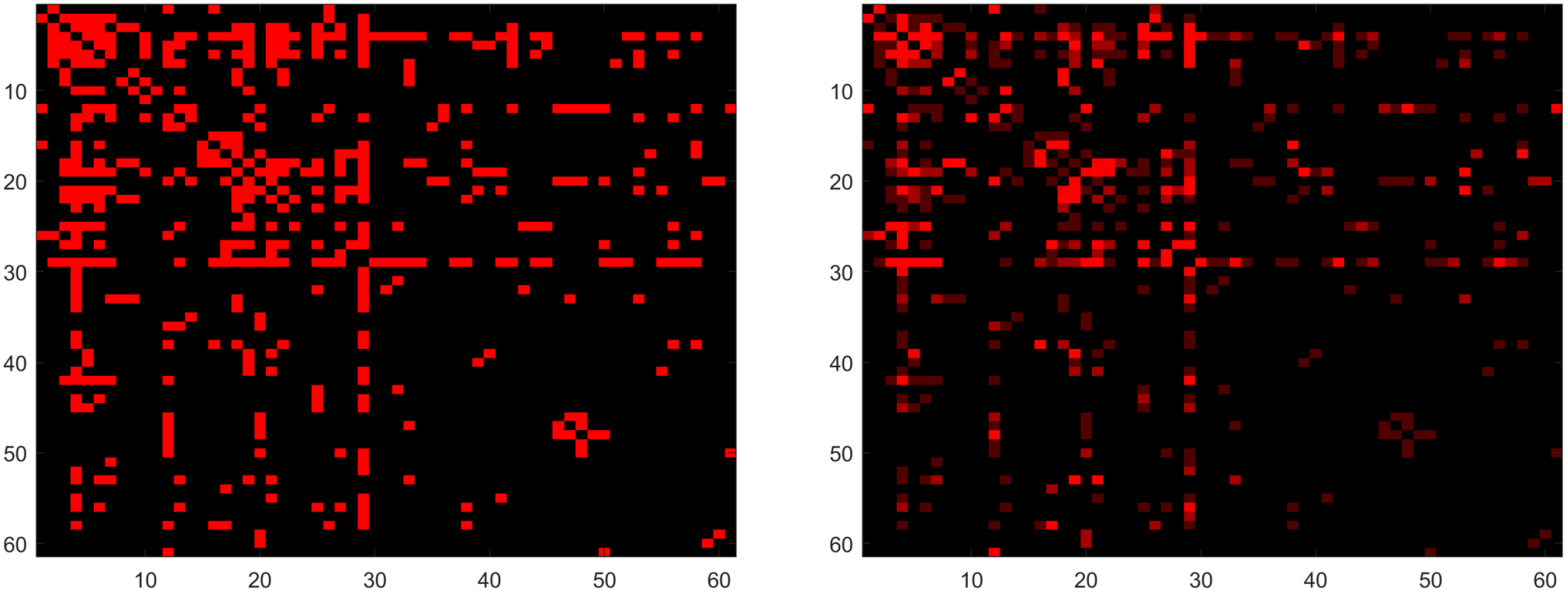
Adjacency matrices from season 1 of *Twin Peaks*: graph (left) and multigraph (right).

The resulting matrix is graphically presented in Figure 5. Our visualization is similar to that of the *Les Misérables* co-occurrence graph [2], though there is a difference in ordering, since we preserve the order of appearance in the matrix. The left representation corresponds to the binary interpretation of the graph seen in Figure 2: characters either share screen time or don’t share it; the amount of time shared is irrelevant. Red squares correspond to edges of the graph, i.e., characters that are connected. On the right, the shade of red changes with respect to the number of scenes characters have in common (i.e., interpretation akin to Figure 3); brighter means more interactions.

**Figure 6.**
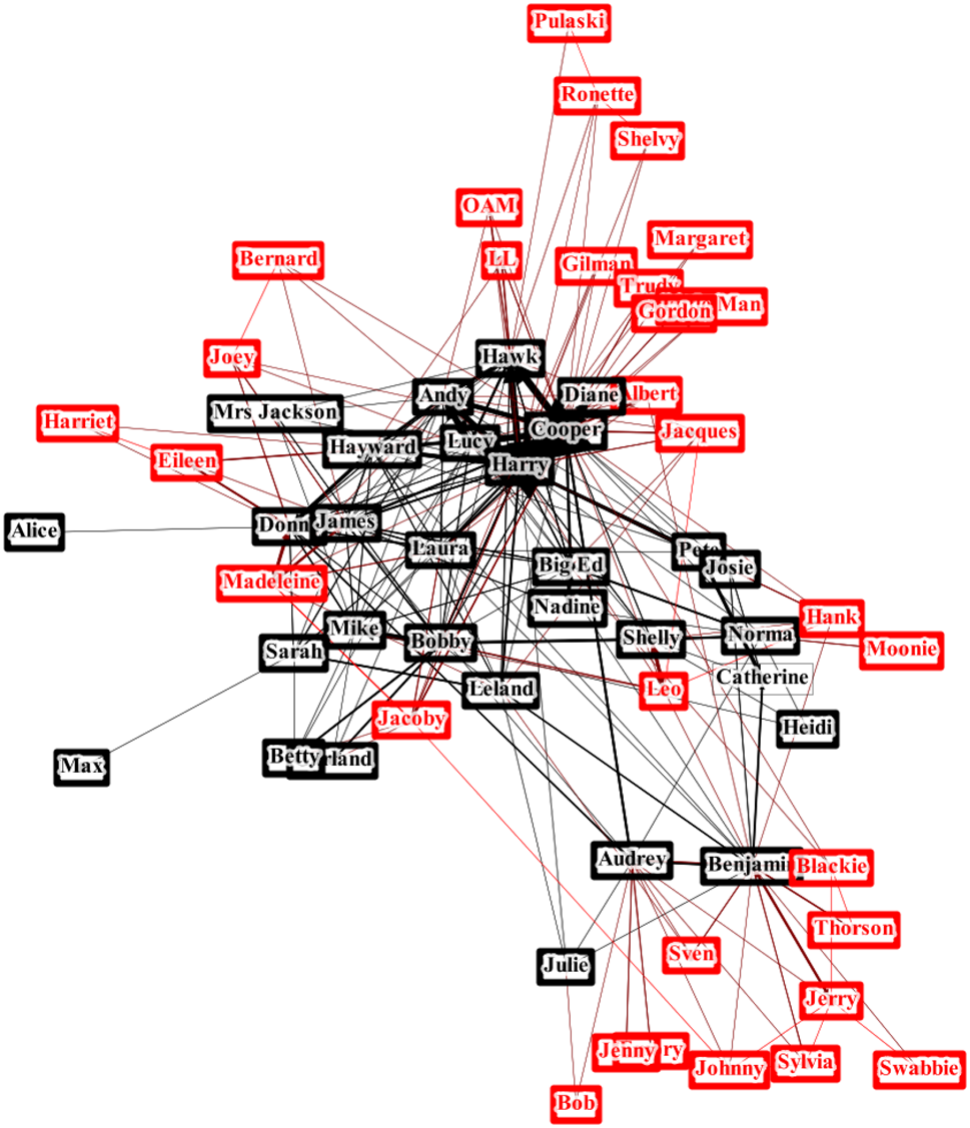
Graphical representation of the *Twin Peaks* network: the black nodes are BC; the red are AD.

We recognize that the characteristic “cross” in the middle of the matrix dividing it into four quadrants represents the protagonist of the show, special agent Dale Cooper. Cooper first appears 36 minutes into the pilot (first episode of the show), and he is the median character: $\approx 30$ characters are introduced before him, and $\approx 30$ after him (as we stated already, the adjacency matrix is filled chronologically, so the *n*th row/column corresponds to the *n*th character to appear in the show). We now divide the characters into two categories—those appearing before Cooper (denoted BC in the remainder of this paper) and those after Dale (AD). The brightest points in the right adjacency matrix are usually either couples or the Twin Peaks Police Department combined with Cooper. Graphically, this is shown in Figure 6, while the most popular nodes and links are tabulated in Table 1.

**Table 1.** Popular links and nodes in the network: all of them are in the BC part.

	The most connected characters	#	The most repeated links	#
1	Dale Cooper	35	Cooper & Truman	47
2	Sheriff Truman	35	Truman & Lucy	16
3	Deputy Andy	20	Cooper & Hawk	15
4	Donna	19	Truman & Hawk	14
5	Bobby	18	Donna & James	13

The most connected AD character appears as the 18th in the list; the top repeated link that includes an AD character appears as the 13th in the list.

**Figure 7.**
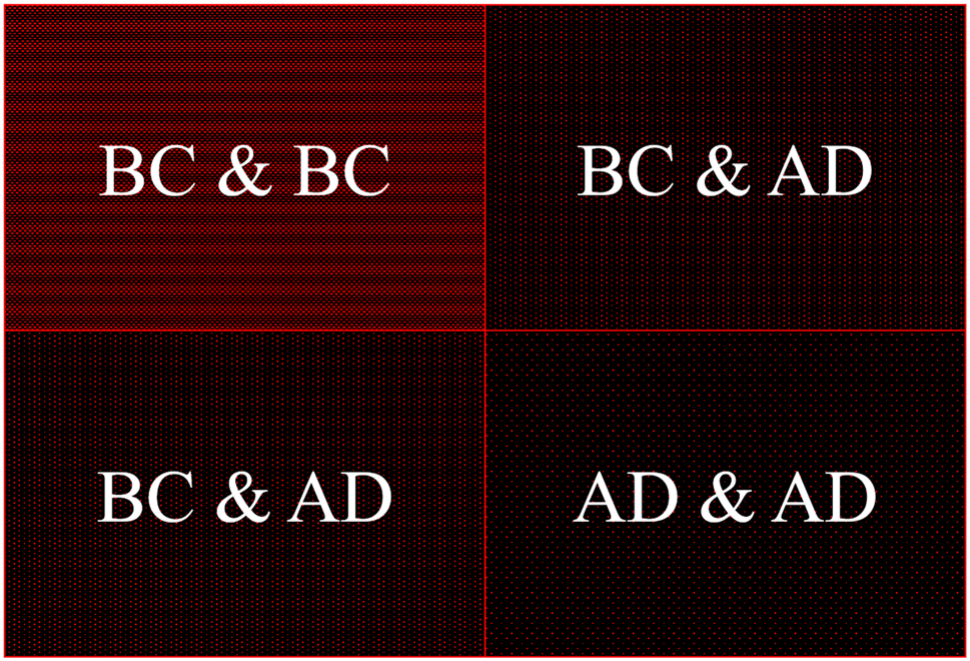
The Dale Cooper effect.

In Figure 7 we discuss the relationship between networks of BC characters and AD characters—what we call the Dale Cooper effect. This cartoon version of the adjacency matrix aims to show how BC is a closely knit community—the part of the adjacency matrix corresponding to it (top left quadrant) is densely filled with connections. On the other end, the AD characters interact among themselves very little, and so the bottom right quadrant is very sparse in connections. It is by virtue of interacting with BC characters that AD characters are a part of the network (bottom left and top right quadrants).

This suggests a mechanism by which the two networks are spliced together. After the nodes of the BC network are set up and Dale Cooper is introduced, new AD nodes are added with a preferential attachment to BC: a newly added node has a much higher probability of being connected to the BC core than to fellow AD periphery. Also, the density of connections is indicative: the full graph has 236 edges, while the BC graph (in this work, we include Cooper in BC) has 128. Doubling the number of characters (i.e., adding AD to BC) hence results in doubling the number of edges (linear scaling).

The mechanism of network growth in the previous paragraph inspires a dive into the degree distribution of the network. Cooper and Sheriff Truman appear together in many scenes and share most of their contacts: their degree is the same, and rather high (35 in the entire network, i.e., more connected with more than half of the other characters; 20 in the BC network, growing the fraction to two-thirds of other characters). Motivated by the findings from *Beowulf* and *Táin Bó Cúailnge*, where adjustments are needed to make the protagonists more realistic, we were curious to see whether such high-degree nodes as those of Cooper and Truman are expected in networks like these.

**Figure 8.**
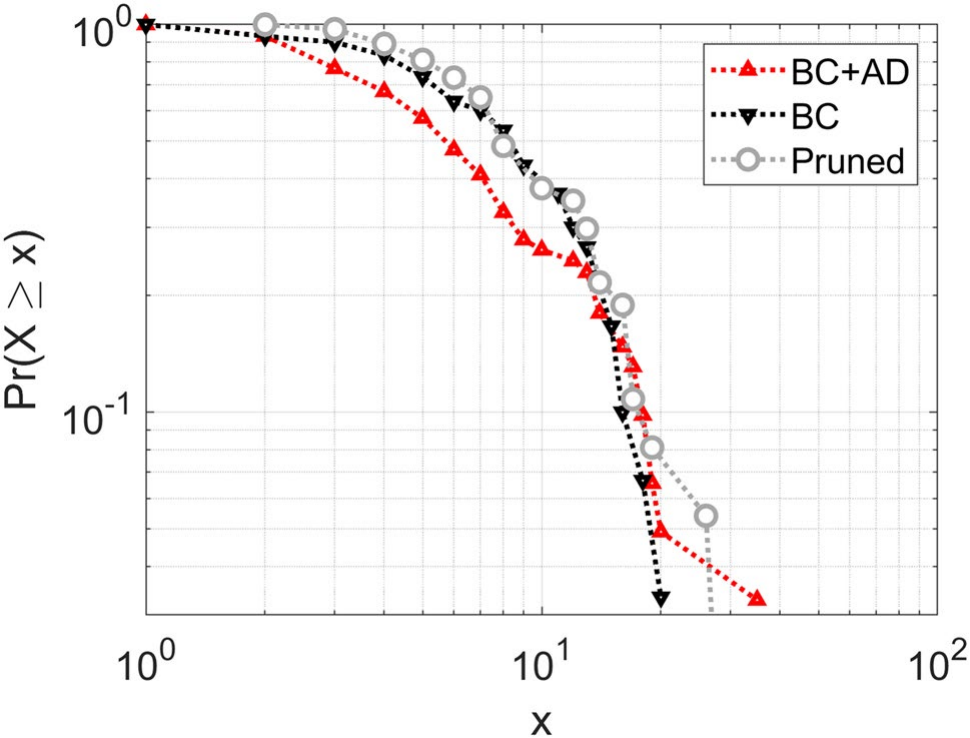
Degree distribution in the *Twin Peaks* networks compared to random networks.

Figure 8 presents our findings. First, we note that the BC degree distribution is similar to that of the random Erdős–Renyi network seen in Figure 4, and not too far off from a log-normal distribution (whose surprising appearance in some preferential attachment networks we already mentioned). For the BC + AD network, we examine significant deviation from the shape: the curve appears to be a superposition of three segments: one for low degrees ($x<10$), one for moderately high degrees ($x<20$), and one for very high degrees ($x>30$). This is the effect of the mechanism by which AD characters are connected: they dominate the low-degree segment of the distribution and shift it leftward while contributing to an increase in degree for Cooper and Truman (the very high degrees data point in the plot), pushing them rightward. From here we deduce that Truman and Cooper are not abnormally well connected in the BC network; but in the BC + AD network, they are more popular than they would be in a random network.

This makes sense, since Truman and Cooper drive the plot in the AD introduction period and are often the first point of contact for new characters. This is mirrored in the assortativity measures for the BC and BC + AD networks: BC assortativity is approximately zero (neutral), while for BC + AD, the assortativity is negative: less well connected characters in AD are connected to popular BC characters.

Following ideas from [5], we proceed with studying the options of character removal. Our first question is about the effect of removing characters that appear in a single scene (correction similar to that applied to *Táin Bó Cúailnge* in [5]). This is the pruned network in Figure 8: the removed nodes are predominantly in the AD group (20 removed from AD and 4 from BC), so it is not surprising that the pruned network gets closer to the BC degree distribution. However, the anomaly of Cooper and Truman remains. As it turns out, the pruned network collapses onto BC if one of the two dominant nodes is removed: a case for “this town ain’t big enough for both of us.” In a way, removing Cooper could be justified as removing the outsider (or Beowulf, following [5]) from the local network of *Twin Peaks*.

Finally, given the importance of the law enforcement characters in a show driven by crime investigation, an interesting test is whether the BC + AD network remains connected after the members of the FBI and the Twin Peaks Police Department are removed. The answer is yes—it still remains a single graph (with the exception of the few characters whose appearance in the show, if limited to Season 1, is tied exclusively to the police investigation), with Benjamin Horne as its central figure, tying together the young population of Twin Peaks through his daughter Audrey, and the criminal milieu through his business connections.

**Figure 9.**
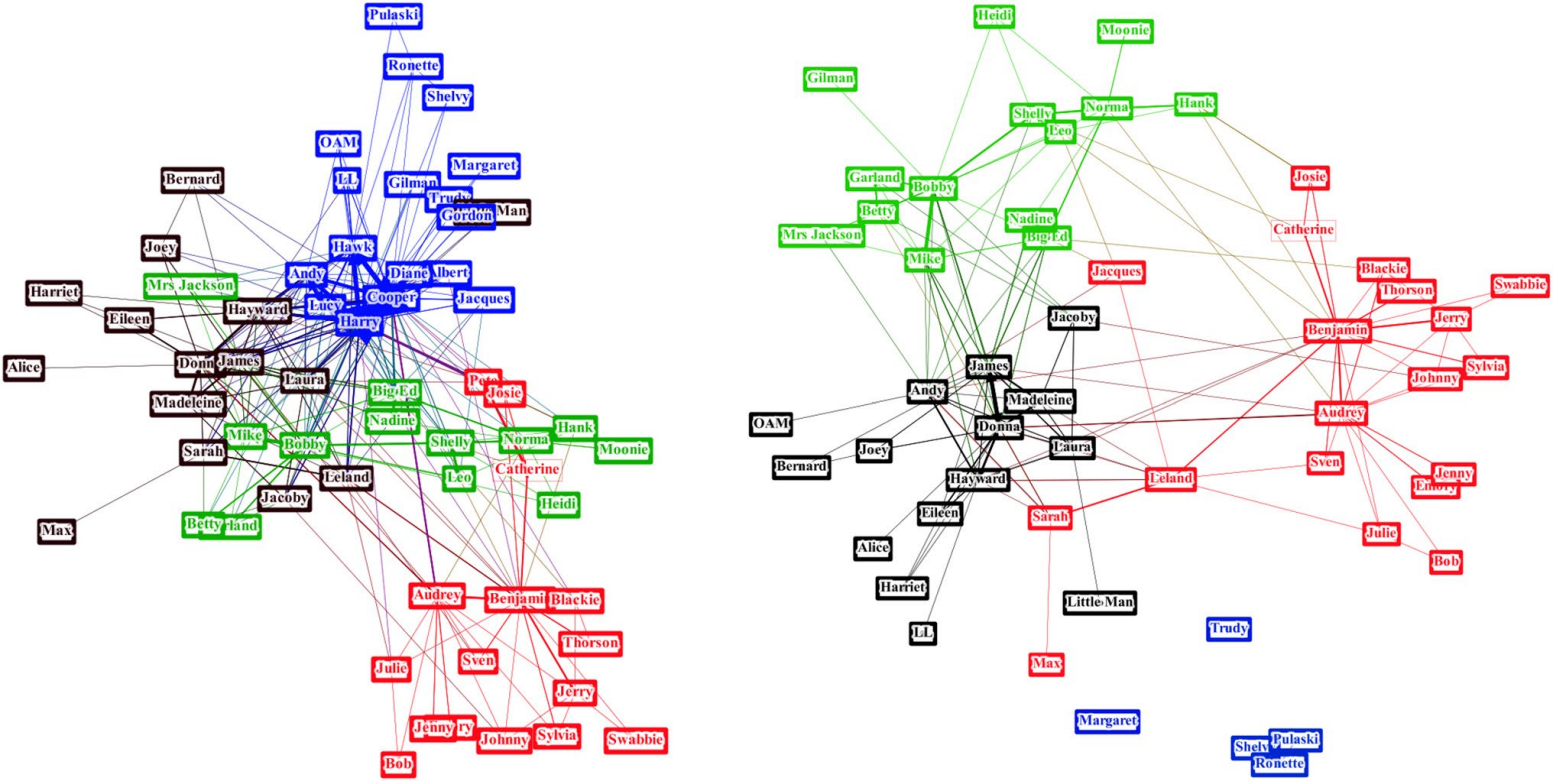
Clusters in the *Twin Peaks* network before (left) and after (right) removing law enforcement.

This process of “police removal” is interesting from the clustering point of view. Networks can often be split into meaningful clusters of nodes based on the connections between them. Sometimes this split is ambiguous, and it is hard to deal with borderline cases, but they give a general idea of the communities existing in the network. Using the Louvain algorithm [7], we perform clustering of the network both before and after removal of police officers in *Twin Peaks*, with the results shown in Figure 9. The blue cluster on the left represents the cluster consisting of the police and people primarily interacting with the police, either as suspects or witnesses. Once the police officers are removed from the network, some nodes are left disconnected, and the rest of the nodes that had connections with other clusters join those clusters.

## Final Remarks

It is hard to draw conclusions from small data sets and somewhat arbitrary conventions applied in its analysis. Given that we are in the realm of art, creation, and interactions, one alternative to the approach taken in this work would have been resorting to the actor network theory (despite the fact that Latour would object to considering the actor network a mathematical network [3]). Is the dead body of Laura Palmer a character? Is the photo of Laura Palmer a character, and if so, is it the same one as the body? How about the Roadhouse?

Furthermore, this study largely ignored the multiplicity of common appearances of characters in scenes. For instance, in our count, Truman and Cooper share almost 50 common appearances, often in scenes that involve other characters as well. This makes them heavily correlated, and they may appear as a single entity, a strongly connected pair. Studying correlations of this sort is a natural extension of the work presented here.

The one takeaway that stands out and presents a statistically robust result is the Dale Cooper effect. The sharp demarcation between the two networks embodied by the protagonist introduced as a median character is a result of the peculiar shape of the story. Do similar structures emerge in other works of fiction? What is the fundamental relationship between the Dale Cooper effect and storytelling? Those questions guide our future work.
